# Characteristics of _L_-citrulline transport through blood-brain barrier in the brain capillary endothelial cell line (TR-BBB cells)

**DOI:** 10.1186/s12929-017-0336-x

**Published:** 2017-05-10

**Authors:** Kyeong-Eun Lee, Young-Sook Kang

**Affiliations:** College of Pharmacy and Research Center for Cell Fate Control, Sookmyung Women’s University, 52, Hyochangwon-gil, Yongsan-gu, Seoul, 140-742 South Korea

**Keywords:** _L_-Citrulline, Blood-brain barrier (BBB), Large amino acid transporter 1(LAT1), Nitric oxide (NO), _L_-Dopa-Gabapentin

## Abstract

**Background:**

_L_-Citrulline is a neutral amino acid and a major precursor of _L_-arginine in the nitric oxide (NO) cycle. Recently it has been reported that _L_-citrulline prevents neuronal cell death and protects cerebrovascular injury, therefore, _L_-citrulline may have a neuroprotective effect to improve cerebrovascular dysfunction. Therefore, we aimed to clarify the brain transport mechanism of _L_-citrulline through blood-brain barrier (BBB) using the conditionally immortalized rat brain capillary endothelial cell line (TR-BBB cells), as an in vitro model of the BBB.

**Methods:**

The uptake study of [^14^C] L-citrulline, quantitative real-time polymerase chain reaction (PCR) analysis, and rLAT1, system b^0,+^, and CAT1 small interfering RNA study were performed in TR-BBB cells.

**Results:**

The uptake of [^14^C] _L_-citrulline was a time-dependent, but ion-independent manner in TR-BBB cells. The transport process involved two saturable components with a Michaelis–Menten constant of 30.9 ± 1.0 μM (Km_1_) and 1.69 ± 0.43 mM (Km_2_). The uptake of [^14^C] _L_-citrulline in TR-BBB cells was significantly inhibited by neutral and cationic amino acids, but not by anionic amino acids. In addition, [^14^C]_L_-citrulline uptake in the cells was markedly inhibited by 2-aminobicyclo-(2,2,1)-heptane-2-carboxylic acid (BCH), which is the inhibitor of the large neutral amino acid transporter 1 (LAT1), B^0^, B^0,+^ and harmaline, the inhibitor of system b^0,+^. Gabapentin and _L_-dopa as the substrates of LAT1 competitively inhibited the uptake of [^14^C] _L_-citrulline. IC_50_ values for _L_-dopa, gabapentin, _L_-phenylalanine and _L_-arginine were 501 μM, 223 μM, 68.9 μM and 33.4 mM, respectively. The expression of mRNA for LAT1 was predominantly increased 187-fold in comparison with that of system b^0,+^ in TR-BBB cells. In the studies of LAT1, system b^0,+^ and CAT1 knockdown via siRNA transfection into TR-BBB cells, the transcript level of LAT1 and [^14^C] _L_-citrulline uptake by LAT1 siRNA were significantly reduced compared with those by control siRNA in TR-BBB cells.

**Conclusions:**

Our results suggest that transport of _L_-citrulline is mainly mediated by LAT1 in TR-BBB cells. Delivery strategy for LAT1-mediated transport and supply of L-citrulline to the brain may serve as therapeutic approaches to improve its neuroprotective effect in patients with cerebrovascular disease.

## Background

_L_-Citrulline is a neutral and non-protein amino acid which was first identified in the juice of watermelon, *Citrullus vulgaris Schrad* [1]. _L_-Citrulline has usually been known as a metabolic intermediate in the urea cycle. Recently, _L_-citrulline has been investigated with a focus on _L_-citrulline as a product of the nitric oxide (NO) cycle and as a precursor for arginine by nitric oxide synthase (NOS) [2, 3]. _L_-Citrulline is converted to _L_-arginine by argininosuccinate synthase and lyase in the NO cycle [4]. As _L_-arginine can be recycled from _L_-citrulline through the NO cycle in some cells such as intestinal cells [5], _L_-citrulline plays an important role in NO metabolism and regulation [3].

In the central nervous system (CNS), NO plays an important role in the cell death or survival mechanisms in brain cells [6, 7]. Neuronal NOS (nNOS) is expressed in neuronal tissues such as neurons and synaptic spines. Inducible NOS (iNOS) can be synthesized by pro-inflammatory cytokines or endotoxin. Endothelial NOS (eNOS) is found in endothelial cells [8]. In general, NO produced by eNOS regulates numerous physiological actions and is neuroprotective to the brain, whereas the comparatively large amount of NO generated by iNOS evokes oxidative stress and is clearly neurotoxic to the brain [9]. nNOS is involved in modulating physiological functions such as learning, memory, and neurogenesis, and pathological condition in the CNS such as Parkinson’s disease and Alzheimer’s disease [10]. Abnormal elevation of NO causes brain damage following cerebral ischemia during the subacute phase [11, 12]. Recently, the neuroprotective effect of _L_-citrulline on CNS disorders such as brain ischemia has been investigated [13]. Previous studies have shown that _L_-citrulline not only prevented neuronal cell death but it also prevented capillary loss in the hippocampal region by cerebral ischemia. The cerebrovascular protective effect of _L_-citrulline was associated with the restoration of endothelial nitric oxide synthase (eNOS) expression in the hippocampus [13]. Thus, _L_-citrulline administration may offer a potential therapeutic strategy not only for patients with impaired arginine metabolism and deficiencies but also for controlling NO metabolism disorders and cell death in the CNS [3, 13].

Neutral amino acids such as _L_-citrulline are transported through cell membranes by several distinct transport systems in different cell types, including macrophages [14], rat aortic smooth muscle cells [15], neural cells [16], bovine aortic endothelial cells [17], and intestinal cells [2]. Systems B^0^ and B^0,+^, as Na^+^-dependent transport systems for neutral amino acids, have been identified [18]. Systems b^0,+^, L, and y^+^L are Na^+^-independent transport systems for neutral amino acids in various cell types [19]. In addition, systems B^0,+^ and b^0,+^have also been found to be related to transport of cationic amino acids in human intestinal epithelial cells [2, 19] and proximal tubular cells [20], respectively. System y^+^L, encoded by y^+^LAT1 and y^+^LAT2, mediates the Na^+^-dependent transport of neutral amino acids as well as the Na^+^-independent transport of cationic amino acids [18]. However, the characteristics of the _L_-citrulline transport system across the blood-brain barrier (BBB) are still unclear. Therefore, the purpose of this study was to characterize the transport system for _L_-citrulline through BBB using the conditionally immortalized rat brain capillary endothelial cell line (TR-BBB cells), as an in vitro model of the BBB [21].

## Methods

### Materials

[^14^C]_L_-Citrulline ([^14^C] _L_-citrulline, 56.3 mCi/mmol) was purchased from PerkinElmer (Waltham, Massachusetts, USA). _L_-dopa and Donepezil hydrochloride were provided by Jeil Co. and Daewoong Co. (Seoul, Korea), respectively. Quinidine was obtained from Tokyo Kasei Kogyo Co. (Tokyo, Japan). _L_-Amino acids were purchased from Sigma-Aldrich (St. Louis, Missouri, USA). All other chemicals and reagents were commercial products of reagent grade.

### Cell culture

The TR-BBB cells established from transgenic rats harboring the temperature-sensitive simian virus 40 large T-antigen, an in vitro BBB model, were cultured at 33 °C as described previously (21). TR-BBB cells were received from Professor Tetsuya Terasaki (Tohoku University, Japan) and were cultured with Dulbecco’s modified Eagle’s medium (Invitrogen, San Diego, CA), supplemented with 10% fetal bovine serum, 100 U/mL penicillin, 100 μg/mL streptomycin (Invitrogen, San Diego, CA) and 15 μg/L endothelial cell growth factor (Roche, Mannheim, Germany) at 33 °C in a humidified atmosphere of 5% CO_2_/air. On rat tail collagen type I-coated 24 well culture plates (IWAKI, Tokyo, Japan) initial seeding was carried out at 1 × 10^5^ cells/well for the uptake study. After incubation for 2 days at 33 °C, the cultures became confluent and then they were used in the transport study. _L_-Citrulline free medium was used all experiments except for experiments on saturation kinetics of [^14^C]_L_-citrulline uptake (Fig. 2), Lineweaver-Burk plots for [^14^C]_L_-citrulline uptake (Fig. 4), or inhibitory effect of _L_-amino acids as a control (Table 2).

### Uptake study in TR-BBB cells

The [^14^C] _L_-citrulline uptake study was performed according to the previous report [22]. Briefly, extracellular fluid (ECF) buffer containing [^14^C]_L_-citrulline (44.4 μM) in the presence or absence of unlabeled inhibitors was added to the TR-BBB cells and then incubated at pH 7.4 and 37 °C for the designated time (5 min). Uptake was terminated by the addition of ice-cold ECF buffer. A Na^+^ free transport medium was prepared by using LiCl, choline chloride, sodium gluconate and KHCO_3_ instead of NaCl and NaHCO_3_, respectively. The cells were then solubilized by incubation overnight in 750 μL of 1 N NaOH at room temperature, and the measurement of radioactivity was performed in a liquid scintillation counter (LS6500; Beckman, Fullerton, CA). Cell to medium ratio (μL/mg protein) was calculated as follows: the radioactivity (dpm/μL) in the sample per milligram cell protein (dpm/mg protein).

### Data analysis

For kinetic studies, the Michaelis-Menten constant (*K*_m_) and the maximum uptake rate (*V*_max_) of [^14^C] _L_-citrulline were estimated from Eq. (1):

1 $$ V={V}_{\max}\cdot C/\ \left({K}_{\mathrm{m}}+ C\right) $$

Where *V* and *C* are the initial uptake rate of [^14^C] _L_-citrulline at 5 min and the concentration of _L_-citrulline, and *V*_max_ is the maximum uptake rate for the saturable component.

To analyze the competitive nature of _L_-dopa and gabapentin for [^14^C]_L_-citrulline uptake, Lineweaver-Burk plots were generated. The inhibitory constant (*K*_i_) was calculated from Eq. (2):

2 $$ V={V}_{\max}\cdot C/\ \left[{K}_{\mathrm{m}}\cdot \left(1 + \mathrm{I}/{K}_{\mathrm{i}}\right) + C\right] $$

where I corresponds to the concentration of _L_-dopa or gabapentin, respectively.

Statistical analyses were carried out by one-way ANOVA with Dunnett’s post-hoc test.

### Preparation of rat cerebrum

An animal experiment was approved by the Committee of the Ethics of Animal Experimentation of Sookmyung Women’s University (SMWU-IACUC-1405-009). Three male Sprague-Dawley (SD) rats (Koatech, Gyeonggi-do, Korea) at aged of 8 weeks (250–350 g) were anesthetized intramuscularly with 100 mg/kg ketamine (Yuhan, Seoul, Korea). After SD rat was anesthetized, the rat was decapitated and the cerebrum was immediately removed. The cerebrum was homogenized with 5 ml syringe (18 gage needle). These homogenized cerebrum tissues (30 mg) were used to isolate total RNA for real-time PCR analysis.

### Real-time PCR analysis

Total RNA was isolated from cultured TR-BBB cells and rat cerebrum tissues by using the RNeasy Mini Kit from Qiagen (Qiagen, Valencia, CA) according the manufacturer’s instructions. Total RNA (2 μg) was reverse-transcribed by using the High Capacity cDNA Reverse Transcription Kit (Applied Biosystems, Life Technologies). Real-time PCR was performed in 48-well plates with the StepOne apparatus (Applied Biosystems, Life Technologies) using the MGB Taqman probe assay. Probes for LAT1, system b^0,+^, CAT1 and endogenous control GAPDH were purchased from Applied Biosystems (Rn00569313_m1, Rn00588400_m1, Rn00565399_m1 and Rn99999916_s1, respectively). Each reaction contained 5 μl Taqman Universal PCR Mastermix in a total volume of 10 μl, and 1 μl cDNA was added to the reaction. Real-time PCR reactions were performed at 50 °C for 2 min, 95 °C for 10 min, followed by 40 cycles of 15 s at 95 °C and 1 min at 60 °C. The results of the analysis were calculated in relation to the GAPDH product, and the results were calculated according to, and expressed by an equation (2^-ΔΔCt^) that gives the amount of target, normalized to an endogenous reference and relative to a calibrator. Ct is the threshold cycle for target amplification (Livak and Schmittgen 2001).

### rLAT1, system b^0,+^, and CAT1 small interfering RNA and small interfering RNA transfection

Transient knockdown of rLAT1, system b^0,+^ and CAT1 in TR-BBB cells was achieved by using small interfering RNA (siRNA) from Dharmacon, GE (Landsmeer, Netherlands). rLAT1, system b^0,+^ and CAT1 were targeted with a SMART pool containing 4 different siRNAs and with each single siRNA individually. The final concentration of siRNA was 200 nM. The rLAT1, system b^0,+^ and CAT1 or control siRNA was delivered individually into TR-BBB cells by using Lipofectamine 2000 (Invitrogen, Carlsbad, CA, USA) according to the manufacturer’s protocol. Cells were used for quantitative real-time PCR and [^14^C]_L_-citrulline uptake was analyzed at 48 h after the initiation of transfection.

## Results

### Characterization of [^14^C]_L_-citrulline transport by TR-BBB cells

**Fig. 1:**
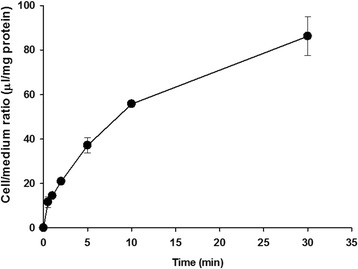
Time-course of [^14^C]_L_-citrulline uptake by TR-BBB cells. [^14^C]_L_-citrulline (44.4 μM) uptake was performed at 37 °C in ECF buffer. Each point represents the mean ± S.E.M. (*n* = 3–4)

To investigate the _L_-citrulline transport system at the BBB, we first performed the [^14^C]_L_-citrulline uptake study using TR-BBB cells, as an in vitro model of the BBB. The uptake of [^14^C] _L_-citrulline was increased in a time-dependent manner and it was linear for 5 min (Fig. 1). Therefore, [^14^C] _L_-citrulline uptake by TR-BBB cells was assessed at 5 min in the subsequent kinetic and inhibition studies. In addition, [^14^C] _L_-citrulline uptake by TR-BBB cells showed no significant difference in the absence of Na^+^ or Cl^−^ in the uptake buffer (Table 1). These results suggested that the transport of _L_-citrulline in TR-BBB cells was mediated by a sodium- and chloride-independent transporter.

**Table 1:** Ion-dependence of [^14^C]_L_-citrulline uptake in TR-BBB cells

Substrate	Uptake of [^14^C]_L_-citrulline (% of control)
Control	100 ± 1.9
Choline Chloride	83.3 ± 5.7
LiCl	116.2 ± 5.2
Sodium gluconate	103.9 ± 6.8

[^14^C]_L_-Citrulline uptake by TR-BBB cells was performed at pH7.4 and 37 °C for 5 min in the presence or absence of sodium and/or chloride. Each value represents the mean ± S.E.M. (*n* = 3–4)

**Fig. 2:**
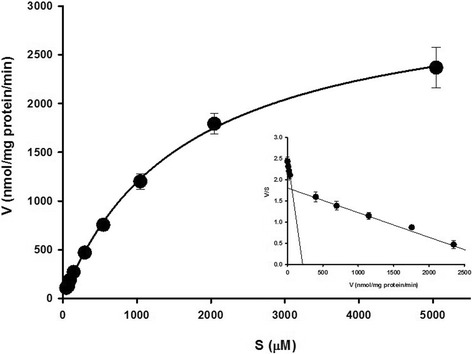
Saturation kinetics of [^14^C]_L_-citrulline uptake by TR-BBB cells. Uptake of [^14^C]_L_-citrulline was measured in TR-BBB cells with 5 min incubation in the presence of 0–5 mM unlabeled _L_-citrulline at pH 7.4 and 37 °C. The data (insert) are shown as an Eadie-Hofstee plot. The values of v and s represent the initial pseudolinear uptake (nmol/mg protein/min) and _L_-citrulline concentration (μM), respectively. The data represent the mean ± S.E.M. (*n* = 3–4)

To analyze the kinetics of [^14^C]_L_-citrulline uptake by TR-BBB cells, we examined the concentration dependence of [^14^C]_L_-citrulline uptake. The transport of [^14^C] _L_-citrulline was saturable (Fig. 2). Kinetic analysis provided two components with a K_m1_ value of 30.9 ± 1.0 μM and a K_m2_ value of 1.69 ± 0.43 mM, which fitted into the Michaelis-Menten equation. In addition, the V_max1_ value was 185 nmol/mg/min, and the V _max2_ value was 3.19 μmol/mg/min. The Eadie-Hofstee plot for [^14^C] _L_-citrulline uptake showed two straight lines, indicating two saturable processes. These data implied that _L_-citrulline transport in TR-BBB cells involved carrier mediated transport system.

### Effect of various _L_-amino acids on [^14^C] _L_-citrulline transport by TR-BBB cells

To examine the _L_-citrulline transport mechanism related to _L_-amino acids in TR-BBB cells, [^14^C] _L_-citrulline uptake was measured in the presence of 2 ~ 20 mM unlabeled _L_-amino acids. The uptake of [^14^C] _L_-citrulline in TR-BBB cells was strongly inhibited by various neutral amino acids such as _L_-phenylalanine and it was significantly inhibited by cationic amino acids such as _L_-arginine and _L_-lysine. Substrates of system ASC such as _L_-alanine, _L_-serine and _L_-cysteine also significantly inhibited [^14^C] _L_-citrulline uptake in the cells. In contrast, anionic amino acids including _L_-glutamate and _L_-aspartate did not inhibit the uptake in TR-BBB cells (Table 2). These results indicated that _L_-citrulline transport in TR-BBB cells is related to neutral and cationic amino acid transport.

**Table 2:** Effect of _L_-amino acids on uptake of [^14^C]_L_-citrulline in TR-BBB cells

Substrate	Concentration (mM)	Uptake of [^14^C]_L_-citrulline (% of control)
Control		100 ± 1.3
_L_-Citrulline	2	36.0 ± 2.1***
_L_-Valine	2	31.5 ± 1.4***
_L_-Leucine	2	49.4 ± 5.8***
	20	17.2 ± 3.9***
_L_-Phenylalanine	2	13.7 ± 1.8***
	20	13.4 ± 1.4***
_L_-Glutamine	2	37.5 ± 1.3***
_L_-Alanine	2	73.0 ± 6.4**
_L_-Serine	2	79.7 ± 2.5*
_L_-Cysteine	2	48.4 ± 2.6***
_L_-Arginine	2	59.9 ± 6.6**
	20	53.2 ± 4.4***
_L_-Lysine	2	74.5 ± 9.0*
	20	66.78 ± 2.1*
_L_-Glutamate	2	102.8 ± 7.8
_L_-Aspatate	2	101.5 ± 8.6

[^14^C]_L_-Citrulline uptake by TR-BBB cells was performed at pH7.4 and 37 °C for 5 min in the presence or absence of 2–20 mM _L_- amino acids. Each value represents the mean ± S.E.M. (*n* = 3–4). *p < 0.05, **p < 0.01, ***p < 0.001; significantly different from control

### Effect of inhibitors of several transporters on [^14^C] _L_-citrulline transport by TR-BBB cells

To identify the transporters involved in _L_-citrulline transport in TR-BBB cells, an inhibition study assessing the effect of several transporter inhibitors on [^14^C] _L_-citrulline uptake was conducted. [^14^C]_L_-citrulline uptake was markedly inhibited by BCH, which is the inhibitor of systems L, B^0^ and B^0,+^. In addition, harmaline, the inhibitor of system b^0,+^ significantly reduced the uptake to 39% of the control. However, there was no inhibition effect of _L_-methylmaleimide, homoarginine, and N-(methylamino) isobutyric acid (MeAIB), which are the inhibitors of systems y^+^L, y^+^, and A, respectively (Table 3). These results implied that _L_-citrulline transport in TR-BBB cells is related to systems L and b^0,+^.

**Table 3:** Effect of several transporter inhibitors on uptake of [^14^C]_L_-citrulline in TR-BBB cells

Substrate	Concentration (mM)	Uptake of [^14^C]_L_-citrulline (% of control)
Control		100 ± 1.3
BCH	2	25.3 ± 4.4***
	20	28.1 ± 11.6***
Harmaline	2	61.0 ± 10.5**
Methylmaleimide	2	103.2 ± 10.1
Homoarginine	2	95.98 ± 13.8
MeAIB	2	99.8 ± 6.3

[^14^C]_L_-Citrulline uptake by TR-BBB cells was performed at pH7.4 and 37 °C for 5 min in the presence or absence of 2–20 mM inhibitors. Each value represents the mean ± S.E.M. (*n* = 3–4). **p < 0.01, ***p < 0.001; significantly different from control

### Inhibition of [^14^C] _L_-citrulline uptake by several drugs in TR-BBB cells

To investigate the transport effect between _L_-citrulline and several drugs in TR-BBB cells, we conducted the inhibition study for [^14^C] _L_-citrulline uptake in TR-BBB cells. _L_-dopa and gabapentin, which are the substrates of system L, strongly inhibited the uptake of [^14^C] _L_-citrulline. In addition, verapamil and quinidine significantly inhibited the uptake of _L_-citrulline. In contrast, donepezil, tacrine, dopamine and riluzole had no effect on [^14^C] _L_-citrulline uptake in TR-BBB cells.

### Inhibitory effect of several _L_-amino acids and drugs on [^14^C]_L_-citrulline transport by TR-BBB cells

**Fig. 3:**
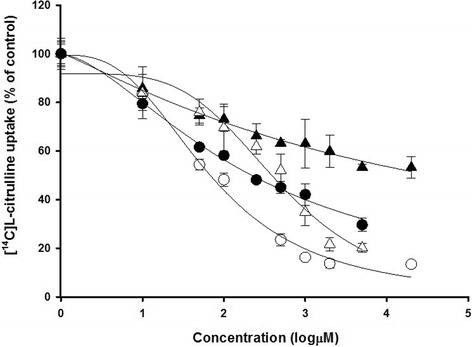
Dose-response relationship for the inhibition of [^14^C]_L_-citrulline uptake by several _L_-amino acids and drugs. Uptake of [^14^C]_L_-citrulline was measured in TR-BBB cells with 5 min incubation in the presence of 0–20 mM unlabeled _L_-phenylalanine (*open circle*) and _L_-arginine (*closed triangle*), 0–5 mM _L_-dopa (*open triangle*) and gabapentin (*close circle*) at pH 7.4 and 37 °C. The data represent the mean ± S.E.M. (*n* = 3–4)

The dose-response relationship for the inhibition of [^14^C]_L_-citrulline uptake by _L_-phenylalanine, _L_-arginine, _L_-dopa and gabapentin in TR-BBB cells is given in Fig. 3. The IC_50_ values for _L_-dopa, gabapentin, _L_-phenylalanine and _L_-arginine were 501 μM, 223 μM, 68. 9 μM and 33.4 mM, respectively.

**Fig. 4:**
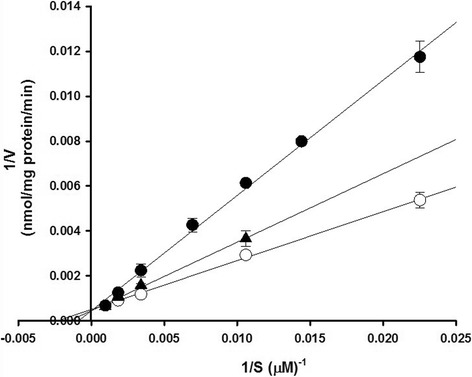
Lineweaver-Burk plots for [^14^C]_L_-citrulline uptake by TR-BBB cells showing competitive inhibition by _L_-dopa and gabapentin. [^14^C]_L_-citrulline (44.4 μM) uptake was performed in the presence of 300 μM _L_-dopa (*close circle*) and 300 μM gabapentin (*close triangle*) or in their absence (*open circle*) in TR-BBB cells at pH 7.4 and 37 °C for 5 min. The data represents the mean ± S.E.M. (*n* = 3–4)

Lineweaver–Burk plot for _L_-citrulline uptake in TR-BBB cells showed the inhibitory effect of _L_-dopa and gabapentin (Fig. 4). [^14^C] _L_-Citrulline uptake in TR-BBB cells was competitively inhibited by _L_-dopa and gabapentin with K_i_ values of 486 μM and 679 μM, respectively.

### Expression of mRNA for LAT1 and system b^0,+^ in TR-BBB cells

**Fig. 5:**
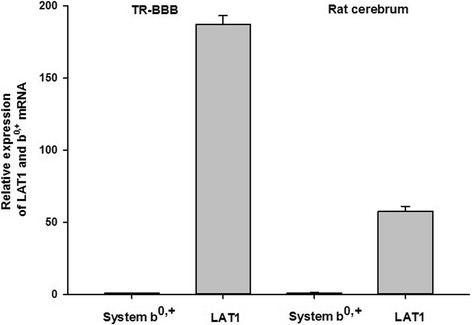
LAT1 and system b^0,+^ mRNA expression was determined by Taqman real-time PCR analysis in the rat cerebrum and in TR-BBB cells. Total RNA (2 μg) was reverse-transcribed and cDNA was amplified by real-time PCR. (+) and (−) represent the presence or absence of reverse transcriptase, respectively. The results were calculated using the comparative Ct (2^−ΔΔCt^) method for relative quantification based on GAPDH mRNA expression and are shown as a fraction of relative LAT1 and system b^0,+^ expression in rat cerebrum and TR-BBB cells. Each value represents the mean ± standard error (SE) of three determinations

To evaluate which transport system was mainly used for _L_-citrulline transport in TR-BBB cells, we performed real-time PCR analysis of the mRNA expression of LAT1 and system b^0,+^ in TR-BBB cells and rat whole brain. The mRNA expression level of LAT1 was about 187 fold higher compared with system b^0,+^ in TR-BBB cells. But, the mRNA expression level of LAT1 was 57.4 fold higher in comparison with system b^0,+^ in rat cerebrum (Fig. 5). These results suggest that LAT1 is mainly involved in _L_-citrulline transport in TR-BBB cells.

### Effects of rLAT1, system b^0,+^ and CAT1 siRNA on transcript levels of rLAT1, system b^0,+^ and CAT1 and [^14^C] _L_-citrulline uptake in TR-BBB cells

**Fig. 6:**
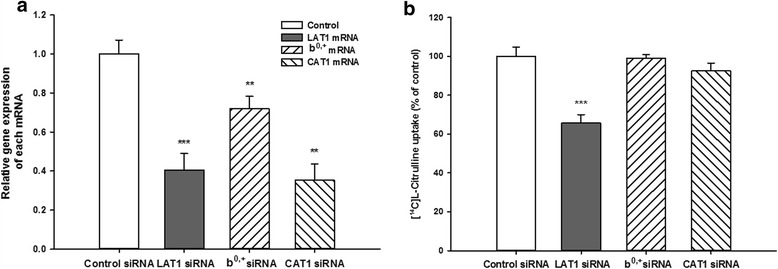
Effect of LAT1, system b^0,+^ and CAT1 siRNA on the expression of each mRNA (**a**) and [^14^C]_L_-citrulline uptake (**b**) in TR-BBB cells. **a** The transcript levels of LAT1, system b^0,+^ and CAT1 were determined by quantitative real-time PCR analysis and normalized to those of GAPDH. **b** [^14^C]_L_-citrulline (44.4 μM) uptake was performed at 37 °C for 5 min. Each column represents the mean ± S.E.M. (*n* = 3–4). **p < 0.01, ***p < 0.001; significantly different from control

In order to confirm whether LAT1, system b^0,+^ and CAT1 were involved in _L_-citrulline transport in TR-BBB cells, we performed rLAT1, system b^0,+^ and CAT1 knockdown by siRNA transfection into TR-BBB cells. The transcript level of each mRNA and [^14^C] _L_-citrulline uptake were determined 48 h after siRNA transfection into TR-BBB cells. The transcript levels of rLAT1, system b^0,+^ and CAT1 were significantly decreased by 60, 28 and 65%, respectively, compared to that of control siRNA on quantitative real-time PCR analysis (Fig. 6a). On the other hand, [^14^C]_L_-citrulline uptake in TR-BBB cells transfected only with rLAT1 siRNA was significantly reduced by 34% compared with that of control siRNA (Fig. 6b), suggesting that LAT1 is mainly involved in [^14^C]_L_-citrulline uptake by TR-BBB cells.

## Discussion

The purpose of this study was to investigate the transport characteristics of _L_-citrulline with use of various compounds and drugs at the BBB. Brain endothelial cells are the main component of the BBB and they express many transporters for substances, such as drugs, chemical compounds, amino acids, and proteins [23]. Due to the different structures and properties of substrates or drugs, it is important to understand the transport system in order to regulate their permeability from blood to brain. In the present study, we used TR-BBB cells which were established as an in vitro model of the BBB by Hosoya et al. [21].

_L_-Citrulline transport has been reported to be mediated by Na^+^ independent and/or Na^+^ dependent transport system in different cell types such as rat intestinal Caco-2 cells, macrophages, etc. [19]. Our results showed that the uptake of [^14^C]_L_-citrulline was a time-dependent (Fig. 1), but Na^+^ and Cl^−^-independent (Table 1) transport occurred in TR-BBB cells. In the kinetic uptake study of [^14^C]_L_-citrulline, _L_-citrulline was transported by two saturable carrier-mediated transport systems (Fig. 2). These data suggested that transport of _L_-citrulline involves Na^+^-independent carrier-mediated transport systems in TR-BBB cells. Regarding the interaction of various amino acids with _L_-citrulline transport in TR-BBB cells, the uptake of [^14^C]_L_-citrulline was strongly inhibited by neutral amino acids and it was significantly inhibited by small neutral amino acids and cationic amino acids (Table 2). However, there were no inhibition effects of several anionic amino acids including _L_-glutamate and _L_-aspartate (Table 2). These results were in accordance with the results obtained in HK-2 cells in the previous study by Mitsuoka et al. [20]. System X^−^_AG_ may not be involved in the transport of _L_-citrulline at the BBB, as _L_-citrulline is in a zwitterionic state at physiological pH [24]. Thus, these data suggested that _L_-citrulline transport is mediated by both neutral amino acid and cationic amino acid transport systems. When we investigated the inhibition effect of candidate inhibitors of several transporters on _L_-citrulline transport in TR-BBB cells, [^14^C]_L_-citrulline uptake was decreased by about 75% with BCH (Table 3). BCH is an amino acid-related compound that has been used as a selective inhibitor of system L including LAT1 and LAT2 [25, 26]. It has also been reported as the inhibitor of systems B^0^ and B^0,+^ [27]. However, systems B^0^ and B^0,+^ are usually related to the Na^+^-dependent transport system for neutral amino acids [18]. Thus, these results indicated that BCH acted as the inhibitor of system L for _L_-citrulline transport in TR-BBB cells. In addition, harmaline, which is the inhibitor of system b^0,+^ [27], significantly inhibited the uptake of [^14^C]_L_-citrulline by about 40% in the cells (Table 3). These data implied that _L_-citrulline transport systems in TR-BBB cells may involve systems L and b^0,+^ as Na^+^-independent transport systems. Previous reports have also mentioned that LAT1 is mainly expressed in bovine brain capillaries [28, 29]. Based on these reports and our results, we performed further studies to compare the mRNA expression of LAT1 with that of system b^0,+^ in TR-BBB cells by quantitative real-time PCR (Fig. 5) in order to investigate which transport system is mostly involved in _L_-citrulline uptake. We confirmed that the mRNA expression level of LAT1 was predominantly increased by about 187 fold compared with that of system b^0,+^ in TR-BBB cells. Also, LAT1 expression was highly increased by 57 fold in comparison with that of system b^0,+^ in rat cerebrum. Moreover, in the functional study of LAT1 and system b^0,+^ knockdown using siRNA transfection, quantitative real-time PCR results showed that the transcript levels of rLAT1 and system b^0,+^ siRNA were significantly reduced compared with that of control siRNA (Fig. 6a), whereas [^14^C]_L_-citrulline uptake by TR-BBB cells transfected with only rLAT1 siRNA was significantly reduced by 34% compared with that of control siRNA (Fig. 6b). Therefore, our finding strongly indicated that LAT1 is mainly involved in _L_-citrulline transport in TR-BBB cells, even though system b^0,+^ is slightly expressed in the BBB. O’Kane RL et al. have reported that harmaline is an inhibitor of system b^0,+^ [27], but it was not considered to be a specific inhibitor of system b^0,+^ only in TR-BBB cells because harmaline has been reported to interact with numerous receptors as well as ion exchangers and voltage-sensitive channels [30]. In addition, based on our results of the inhibition study with _L_-arginine (Table 2.), we also confirmed whether the cationic amino acid transporter 1 (CAT1) is involved in _L_-citrulline transport in TR-BBB cells by performing CAT1 siRNA transfection (Fig. 6). CAT1 has been reported to be the main _L_-arginine transporter in the BBB [31]. CAT1 transports such basic amino acids, and its expression is concentrated in brain capillaries [32]. The uptake study of [^14^C] _L_-citrulline showed that there was no significant reduction by CAT1 siRNA when compared with that by control siRNA in TR-BBB cells (Fig. 6b). These results implied that CAT1 is less relevant for _L_-citrulline transport in TR-BBB cells.

On the other hand, the K_m_ values of LAT1 show about 10 ~ 100 μM of high affinity and 1 ~ 10 mM of low affinity in the BBB [28, 33]. Actually, the K_m_ values of _L_-citrulline (K_m1_ = 30.9 μM and K_m2_ = 1.69 mM) in TR-BBB cells were in the similar range as the values of LAT1 in previous studies. _L_-Arginine significantly inhibited the uptake of [^14^C]_L_-citrulline (Table 2) and the IC_50_ value of _L_-arginine was 33.4 mM (Fig. 3) in TR-BBB cells. Especially, the reason why co-treatment with _L_-arginine inhibited the transport of _L_-citrulline can be considered to be the strong interaction between _L_-arginine and _L_-citrulline due to their structural similarity [2]. In our results, the IC_50_ value of _L_-arginine was relatively high compared with those of _L_-phenylalanine, _L_-dopa and gabapentin. These results implied that _L_-arginine is transported by a different transport system such as CAT1 and system b^0,+^. Moreover, _L_-arginine transport may have a negligible effect on _L_-citrulline transport in clinical conditions due to the high IC_50_ value with a millimolar (mM) level for _L_-arginine in this study. It has been reported that _L_-citrulline has better absorption and systemic bioavailability than _L_-arginine [34, 35] and it did not induce osmotic diarrhea at high dosage compared with _L_-arginine [36]. Also, if there is a different transport system for _L_-arginine as shown by our results, it can be considered that _L_-citrulline treatment is a more effective therapeutic method for _L_-arginine deficiency in clinical conditions. In addition, Shen LJ et al. have reported that argininosuccinate synthase (AS) activity plays a pivotal role in intracellular citrulline-arginine regeneration via eNOS for NO production [37]. Therefore, to elucidate the clinical effect for NO pathway related to _L_-citrulline transport in the BBB, further studies are remained to measure several parameters in NO pathway such as AS, NOS proteins and NO etc. related to _L_-citrulline transport in TR-BBB cells.

In the inhibition study between _L_-citrulline transport and CNS-acting drugs such as donepezil, tacrine, dopamine and riluzole in TR-BBB cells, donepezil and tacrine had no significant inhibition effect on [^14^C] _L_-citrulline uptake (Table 4). Donepezil and tacrine, which are AChE inhibitors and have been used as therapeutic agents for Alzheimer’s disease (AD), show a relatively high distribution in the brain [38–40]. These drugs show a competitive inhibition of choline transport via OCT2 in TR-BBB cells [41] and they are transported across the BBB to the brain via the choline transport system, CHT1 [42, 43]. Due to the use of this transport system for donepezil and tacrine in the BBB, it seems that these drugs do not have inhibition effect for _L_-citrulline transport in TR-BBB cells. We also confirmed that dopamine, riluzole and taurine had no significant effects on [^14^C] _L_-citrulline uptake in TR-BBB cells. Dopamine is a neurotransmitter belonging to the family of catecholamines, and it is a therapeutic agent for Parkinson’s disease (PD) in the brain. In the BBB, dopamine is transported by rat plasma membrane monoamine transporter (rPMAT) in TR-BBB and TR-CSFB cells [44]. Transport of taurine, a beta-amino acid and has neuroprotective effect, which is mediated by TAUT in TR-BBB cells [22]. Riluzole (2-amino-6-trifluoromethoxy benzothiazole) is a neuroprotective drug approved for amyotrophic lateral sclerosis [45] and activates GLT-1 and GLAST to enhance glutamate uptake [46, 47], but there have been poor mechanistic experiments to transport riluzole across the BBB to the brain. _L_-Citrulline transport is not affected by these two drugs via different transport systems in TR-BBB cells. However, _L_-dopa, gabapentin, verapamil, and quinidine significantly inhibited the uptake of [^14^C]_L_-citrulline (Table 4). It has been reported that _L_-dopa and gabapentin are transported across the BBB by LAT1 [48, 49]. Therefore, we hypothesized that the transport systems for _L_-citrulline might involve mainly LAT1. The IC_50_ values for _L_-dopa and gabapentin were 501 μM and 223 μM, respectively (Fig. 3) and they competitively inhibited _L_-citrulline uptake with 486 μM and 679 μM as the K_i_ values for _L_-dopa and gabapentin, respectively in the Lineweaver-Burk plot analysis (Fig. 4). These results indicated that _L_-citrulline may also be a substrate of LAT1 in TR-BBB cells, as _L_-dopa and gabapentin compete with _L_-citrulline for the same binding site on LAT1. However, the maximal plasma concentrations (C_max_) of _L_-dopa and gabapentin at steady state were 1 ~ 20 μM and 23 ~ 80 μM, respectively [50, 51]. These results demonstrated that the K_i_ values for _L_-dopa and gabapentin are several times higher than their C_max_. Therefore, these drugs may not significantly inhibit _L_-citrulline transport via LAT1 at the BBB in clinical conditions. Kageyama et al. reported that _L_-dopa is transported by LAT1 in MBEC4 cells [48]. However, there was no inhibition effect on _L_-dopa transport with cationic amino acids including _L_-arginine and _L_-lysine in their study. These results were not in agreement with our results for _L_-citrulline transport in TR-BBB cells. Therefore, for _L_-citrulline transport across the BBB, the effects of cationic amino acids, not _L_-dopa and gabapentin in TR-BBB cells should be further studied. Furthermore, _L_-citrulline transport across the BBB was also inhibited by CNS-acting drugs such as _L_-dopa and gabapentin via LAT1. Thus, to understand the detailed mechanism of the transport system in therapeutics for CNS disorders across the BBB, further studies related to amino acid transport systems are required.

**Table 4:** Effect of several drugs on uptake of [^14^C]_L_-citrulline in TR-BBB cells

Substrate	Concentration (mM)	Uptake of [^14^C]_L_-citrulline (% of control)
Control		100 ± 1.3
_L_-Dopa	0.5	52.1 ± 6.6**
	2	21.6 ± 2.8***
Gabapentin	0.5	45.0 ± 2.52***
	5	29.6 ± 2.73***
Verapamil	0.5	30.9 ± 0.3***
Qunidine	0.5	52.5 ± 3.0***
Dopamine	2	110.5 ± 2.7
Donepezil	2	85.5 ± 5.9
Riluzole	2	93.9 ± 8.3
Taurine	2	90.5 ± 4.4

[^14^C]_L_-Citrulline uptake by TR-BBB cells was performed at pH7.4 and 37 °C for 5 min in the presence or absence (control) of 2 mM drugs (except for 500 μM quinidine, verapamil and 200 μM of riluzole). Each value represents the mean ± S.E.M. (*n* = 3–4). **p < 0.01, ***p < 0.001; significantly different from control

## Conclusions

Our results demonstrated that _L_-citrulline transport might be mainly mediated by LAT1 in TR-BBB cells. Understanding the transport characteristics of _L_-citrulline to the brain through BBB might contribute to the transport strategy for _L_-citrulline as a potential therapeutic agent for cerebrovascular diseases such as brain ischemia.
